# Mannosylated fisetin/carveol lipid nanocapsules: brain-targeted dual therapy for modulation of epileptogenesis and cognitive deficits

**DOI:** 10.1007/s13346-025-01937-2

**Published:** 2025-08-09

**Authors:** Julie R. Youssef, Nabila A. Boraie, Fatma A. Ismail, Basant A. Bakr, Eman A. Allam, Mahmoud A. Agami, Riham M. El-Moslemany

**Affiliations:** 1 Department of Pharmaceutics, Faculty of Pharmacy, Alexandria University, Alexandria, 21521 Egypt; 2 Department of Zoology, Faculty of Science, Alexandria University, Alexandria, 21523 Egypt; 3 Department of Medical Physiology, Faculty of Medicine, Alexandria University, Alexandria, 21131 Egypt; 4 Faculty of Pharmacy, New Valley University, New Valley Governorate, Egypt; 5 Research and Innovation Hub, Alamein International University, New Alamein city, Egypt

**Keywords:** Phytomedicine, Nanocarrier, Kindling, Brain-derived neurotrophic factor, Glucose transporter, Pentylenetetrazol

## Abstract

Pharmacological treatment of epilepsy is challenged by several barriers with the blood brain barrier (BBB) imposing the utmost restrictions to brain drug delivery. Antiepileptic drugs aim to reduce seizures frequency and severity while exerting minimal toxic effects. Herein, the merits of phytomedicine and brain targeted nanocarriers were combined for the control of seizures in a chronic epilepsy model. Fisetin (FS); a polyphenol and carveol (CAR); a limonene monoterpenoid were selected for their neuroprotective roles and co-loaded into lipid nanocapsules (FS/CAR@LNC). This was further decorated with mannose by post insertion targeting glucose transporter (GLUT-1) overexpressed on BBB (MAN-FS/CAR@LNC). The optimized MAN-FS/CAR@LNC revealed good colloidal properties with particle size (53.17 ± 4.06 nm), and low polydispersity index (0.11) and 26.9 ± 1.05 mV zeta potential. A high entrapment efficiency exceeding 99% and sustained drug release profile over 72 h were observed for both FS and CAR. In the in vivo imaging system (IVIS), MAN-FS/CAR@LNC attained 1.3-fold increase in fluorescence intensity at 5 h interval compared to FS/CAR@LNC. Following intraperitoneal administration in PTZ- induced chronic epilepsy mouse model, MAN-FS/CAR@LNC achieved maximal control of epileptic seizures accompanied with rehabilitation of locomotion, depressive and anxiety like-behaviors compared to FS/CAR and FS/CAR@LNC. Similarly, analysis of biomarkers reflecting depression and anxiety actions (brain-derived neurotrophic factor; BDNF, serotonin and glutamate) together with inflammatory markers (IL-6 and IL-1ꞵ) and histopathological assessment affirmed MAN-FS/CAR@LNC excelling in enhancing LNC brain targetability and hence seizures control. In conclusion, FS/CAR co-therapy aided with mannosylated LNC could present a compelling podium for the effective management of CNS disorders.

**Supplementary Information:**

The online version contains supplementary material available at 10.1007/s13346-025-01937-2.

## Graphical Abstract

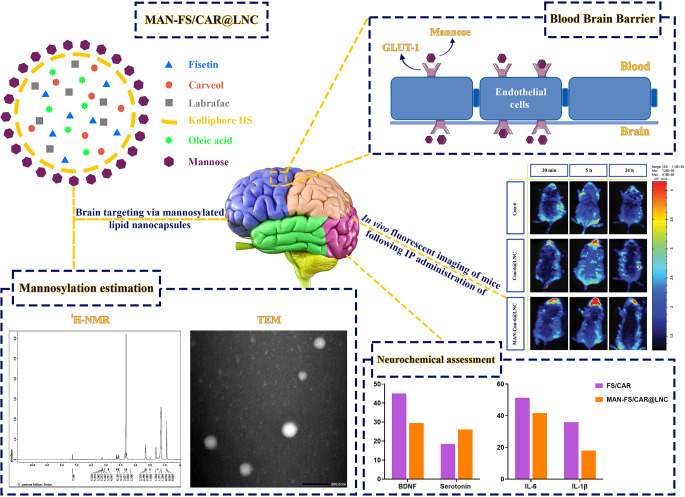

## Introduction

Epilepsy is a neurological disorder delineated by aberrant electrical discharges and neuronal excitability from brain areas manifesting epileptic seizures [1, 2]. The cerebral cortex and hippocampus are the brain areas affected by the hypersynchronous discharges during seizures resulting in molecular and morphological changes in these epileptogenic areas [3]. Epileptic seizures are accompanied by an imbalance between excitatory and inhibitory neurotransmitters; either by boosting of excitatory glutamate neurotransmission and/or deduction of inhibitory gamma-aminobutyric acid (GABA) [4]. Chronic epilepsy triggers the neuroinflammatory cascade through boosting the levels of inflammatory cytokines [5] and the oxidative pathway generating reactive oxygen species [6]. Also, brain-derived neurotrophic factor (BDNF), a neurotrophins protein family member, is crucial for CNS and neuronal plasticity development [7]. Fluctuations of BDNF; increasing or decreasing were shown to affect epilepsy pathogenesis in different ways, however the molecular mechanism is still not clear [7].

Antiepileptic drugs aim to control both frequency and severity of seizures. Despite of their efficacy, current treatments cause prominent side effects [8]. A major barrier facing antiepileptic drugs is their entry into astrocytes. This is challenged by the non-fenestrated structure of the blood brain barrier (BBB) [9] which constitutes endothelial cells mingled with tight junctions conferring regulatory mechanisms controlling the passage of molecules into the brain [10]. During epileptogenesis, alterations in the BBB structure allows for reciprocating entry of large protein particles and blood cells to the brain [11]. It is also accompanied by an up-regulation of the drug efflux transporter; P-glycoprotein (P-gp) [12, 13] leading to drug resistance. Moreover, during epilepsy, therapeutic agents become entrapped in pathologically enlarged perivascular spaces instead of reaching neural targets [14].

Over the last decades, nanotechnology has become a key focus for enhancing antiepileptic drug delivery to the brain circumventing several obstacles facing drug transport across the BBB. In this setting, nanocarriers such as liposomes and polymeric or lipid nanoparticles have previously demonstrated augmented antiepileptic effects of several drugs in preclinical chronic epilepsy models [13, 15].

Lipid nanocapsules (LNC) are physically stable biomimetic nano vectors consisting of lipid core stealth coated by lecithin and a pegylated surfactant tensioactive membrane [16]. LNC are said to integrate the physicochemical and biological properties required for nanocarrier mediated CNS delivery [17]. Their size ranges from 25 to 100 nm with narrow dispersity [18]. The pegylated shell allows for prolonged circulation thus promoting high drug concentration at its specific target [19]. Also, the down regulation of the multi drug resistance transporter; P-gp efflux pump overexpressed in diseased BBB [20] displays a pivotal role in boosting drug bioavailability in the brain [21]. LNC grant definable active targetability via surface modification with different biochemically active groups [16, 22] which is a strategy facilitating brain specific drug delivery via active-targeting, transporter mediated, and receptor mediated transcytosis pathways [23]. Transporter mediated transcytosis demonstrated faster and enhanced efficiency when compared to receptor mediated transcytosis [24]. The high-density expression of glucose transpoter-1 (GLUT-1) normally required for glucose uptake in the brain [25] can be considered as an attractive target. In this context, mannose (MAN) being a specific substrate to GLUT-1 was utilized for coating nanoparticles upsurging BBB targeting [25] thus enhancing brain delivery and hence efficacy. This has been demonstrated for improved BDNF gene therapy following mannosylation of liposomes in rescuing Alzheimer’s disease pathology [26]. Also, mannose functionalized liposomes for brain targeted vgf gene therapy showed reinforced cellular penetration and uptake by brain endothelial cells [25].

An alternative approach shown to be promising in overcoming conventional antiepileptic drugs drawbacks is the use of phytomedicine for seizures control [27]. Fisetin (FS) is a bioactive flavonoid most abundant in strawberries [28] with well documented neuroprotective effect in a myriad of neurological disorders including epileptic seizures [29]. FS administration allowed for hippocampal neuronal damage restoration in lipopolysaccharide-induced neurodegeneration in mice [30]. It was shown to protect against traumatic brain injury, promote neural cell differentiation and inhibit amyloid beta-induced neuroinflammation and synaptic distortion in mice brain hippocampal region [31]. FS also acts by suppressing NF-kB and COX-2 expression in the hippocampus and cortex thus conferring anti-inflammatory function contributing to its neuroprotective action in epileptic mice. Combination therapy via embodying bioactive essential oils or their components to herbal drugs allows for synergistic neurological effects [22]. Carveol (CAR), a natural monoterpene phenol present in the essential oil of dill, orange peel and caraway seeds [32] has been addressed in traditional Chinese medicine for its anti-spasmodic, carminative, astringent role [33]. In CNS disorders, CAR was shown to exert a neuroprotective role in ischemic brains diminishing the infarction area [34] and to attenuate cognitive deficits and neuroinflammation in pentylenetetrazol (PTZ)-induced chronic epilepsy model via Nrf2 antioxidant pathway activation [35].

Herein, the current work aimed to investigate the potential of FS/CAR co-therapy and the role of their LNC loading on their antiepileptic effect. Furthermore, scrutinization of the inherent brain targeting capability of mannosylated LNC (MAN-FS/CAR@LNC) was also carried out. Enhanced brain accumulation was confirmed by in vivo imaging of fluorescently labelled LNC. Furthermore, profound investigation of antipileptic efficacy in a PTZ-induced chronic epilepsy model following intraperitoneal administration via behavioral assessment of locomotor activity, depression and anxiety like behaviors and memory was executed. Also, biochemical markers reflecting inflammation, depression and anxiety in addition to histopathological findings and biocompatibility were tested.

## Materials and methods

### Materials

Fisetin (3, 3’, 4’, 7-tetrahydroxyflavone) (FS) was obtained from Arctom Scientific (Agoura Hills, CA, USA). (-)- Carveol oil (mixture of isomers) (CAR), Oleic acid (OA), Pentylenetetrazol (PTZ), D-(+)-Mannose, Coumarin-6 (Cou-6) and Octadecylamine (stearyl amine) were purchased from Sigma-Aldrich, St. Louis, MO, USA. Kolliphor HS 15 (free polyethylene glycol 660 and polyethylene glycol 660 hydroxystearate mixture, *European Pharmacopeia*, IVth, 2002) was from BASF (Ludwigshafen, Germany). Labrafac^®^ lipophile WL 1349 (caprylic-capric acid triglycerides; *European Pharmacopeia*, IVth, 2002) was a kind gift from Gattefossé SA (Saint-Priest, France). Centrifugal concentrators (Vivaspin6™, MWCO 100,000, Sartorius™). HPLC grade acetonitrile and formic acid were purchased from Thermo Fisher Scientific (Waltham, MA, USA). Commercial Creatinine (Cr) kit was from Genesis (Cairo, Egypt). Commercial blood urea nitrogen (BUN) kit was purchased from Spectrum Diagnostics (Cario, Egypt). Commercial serum aspartate aminotransferase (AST) and alanine aminotransferase (ALT) kits were from VITRO SCIENT (Cario, Egypt). All other reagents were of analytical grade.

### Preparation of blank and drug loaded lipid nanocapsules

LNC were formulated by the solvent free phase inversion method based on oil/water emulsion temperature cycling [16]. Succinctly, a primary emulsion was prepared by mixing Kolliphor HS15 (500 mg), labrafac lipophile (500 mg) under magnetic stirring with deionized water maintaining a ratio of 1:1:3 w/w. Also, NaCl (0.44% w/v, 44 mg in 10 mL dispersion) was added to the mixture and sequentially heated over the phase inversion temperature (PIT) followed by gradual cooling. Owing to FS instability at high temperature [36], oleic acid (OA, 1.5% w/v of the final dispersion volume, 150 mg in 10 mL dispersion) was added to the formulation in order to reduce the PIT and hence the heating/cooling cycle temperature from (65 to 85 °C) to (35–48 °C) [37, 38]. Three heating/cooling cycles were performed followed by a sudden irreversible shock by 4-fold dilution using cold deionized water maintained at a 0–2 °C temperature. Finally, slow magnetic stirring was done for 5 min at room temperature.

Drug loaded formulations were prepared by mixing 1–2 mg/mL of the final dispersion volume of either FS (FS@LNC) or a combination of FS and CAR (FS/CAR@LNC) with the oil/surfactant mixture by stirring for 5 min followed by addition of deionized water. LNC were then prepared using the aforementioned procedure.

### Brain targeted lipid nanocapsules

#### Preparation of mannosylated lipid nanocapsules (MAN@LNC)

For mannose (MAN) functionalization, LNC were modified by incorporation of stearylamine (SA) in the primary emulsion in the ratio 1: 50 and 1: 25 of the oily phase (labrafac). Mannosylation was performed based on a previously reported method for preparation of mannosylated solid lipid nanoparticles [39] with some modifications. Briefly, (MAN) was dissolved in acetate buffer pH 4.5 adjusted with HCL 1 M at a concentration of 100 mM. MAN solution was then added dropwise to the preformed LNC dispersion to achieve different molar ratios of SA: MAN (1:5 and 1:10) followed by stirring for 72 h at room temperature to allow maximum conjugation of MAN to the primary amine groups of SA [40]. Dialysis against double distilled water for 1 h (Visking 36/32, MWCO 12–14 KDa, 28 mm, Serva, Heidelberg, Germany) was used to remove unreacted MAN.

#### Fourier transform infrared spectroscopy (FTIR)

MAN, SA and freeze-dried MAN@LNC with 1:5 SA: MAN molar ratio were scanned using a diamond ATR spectrophotometer (Cary 630, Agilent Biotechnology, Penang, Malaysia) equipped with a horizontal attenuated total reflectance with a diamond crystal as sampling accessory. Spectra in the range 4000 to 600 cm^− 1^ with a 4 cm^− 1^resolution were obtained.

#### Proton nuclear magnetic resonance (^1^H-NMR)

^1^H-NMR spectroscopy of the selected MAN@LNC formulation was investigated using NMR spectrometer (JEOL FCA-500 II) at frequency 500 MHz. A deprotonated solvent CDCl_3_ was used to dissolve the sample.

### Fluorescently labeled LNC

Fluorescently labelled LNC were prepared by encapsulation of Coumarin-6 (Cou-6). Cou-6 was added to the initial mixture in the concentration 0.5 mg/g (Cou-6/labrafac weight ratio) [41] prior to temperature cycling followed by the same procedures as mentioned in the section “Preparation of blank and drug loaded lipid nanocapsules” (Cou-6@LNC) and 2.3.1. (MAN-Cou-6@LNC).

### Physicochemical characterization of LNC

#### Colloidal properties

LNC formulations were analyzed for Z-average particle size (PS) and polydispersity index (PDI) by dynamic light scattering (DLS) technique using a Malvern Zetasizer^®^ at 25 °C at a173° fixed angle using a 4 mW He-Ne (Zetasizer^®^ Nano ZS series DTS 1060, Malvern Instruments S.A, Worcestershire, UK). Also, zeta potential (ZP) was measured using a 150 V cell voltage and a current of 5 mA at 25 °C. Sample dilution with filtered deionized water in the ratio 1:50 v/v was done prior to analysis (*n* = 3).

#### Microscopical examination

LNC and MAN@LNC morphological features were assessed using transmission electron microscopy (TEM) (Jeol electron microscope, JEM-100 CX, Tokyo, Japan). Following diluted with filtered deionized water (1:9 v/v), LNC dispersions were mounted on carbon coated copper grids and stained negatively by a 2% w/v aqueous uranyl acetate solution for 30 s. This was followed by air-drying of samples under ambient conditions then images were taken at x 40 K magnification.

#### Entrapment efficiency and drug payload

FS or CAR % entrapment efficiency (%EE) in FS@LNC, FS/CAR@LNC and MAN-FS/CAR@LNC was assessed using ultrafiltration/centrifugation technique by calculating the free FS or CAR concentrations in the filtrate [37]. Vivaspin^®^ 6 concentrators (MWCO = 100,000, Sartorius, USA) were used. Briefly, 3 mL samples were centrifuged at 6 K rpm at 4 °C for 15 min (Sigma 3-30KS, Sigma Laborzentrifugen GmbH, Germany). Free FS or CAR concentration in the filtrate was analyzed by LC-MS-MS. The chromatographic system consisted of a Thermo Fischer Vanquish Horizon UHPLC system combined with TSQ Fortis™ Plus Triple Quadrupole Mass Spectrometer and TraceFinder™ 5.1 software, the column used for separation was Hypersil GOLD™ (5µ, 150 × 4 mm). Analysis was done at the Advanced Instrumental Analysis Division, The Research and Innovation Hub, Alamein International University. Chromatographic separation was achieved using an isocratic flow of a mixture of 70% acetonitrile in 0.1% formic acid aqueous solution with a 0.8 mL/min flow rate. The column and autosampler temperatures were maintained at 30 °C and 9 °C respectively.

The %EE was measured from the difference between the theoretical drug concentration and the unentrapped drug concentration recovered in the filtrate as in Eq. 1:

1 $$\:EE\%=\frac{total\:drug\:amount-unentrapped\:drug\:}{total\:drug\:amount\:}*100$$

Measurements were done in triplicate (*n* = 3).

Moreover, drug payload (DL) was evaluated respective to the total LNC formulation dry weight (Eq. 2):

2 $$\:DL=\frac{FS\:entrapped\:\left(mg\right)}{Total\:dry\:weight\:\left(g\right)}$$

#### Drug release

The in vitro release profile of FS and CAR from both LNC and MAN@LNC was investigated using ultrafiltration/ centrifugation technique. Samples used were 1 mg/mL of both FS and CAR. Samples (100 µL) of FS@LNC, FS/CAR@LNC and MAN-FS/CAR@LNC equivalent to 0.1 mg FS and 0.1 mg CAR were diluted to 5 mL with PBS of pH 7.4 containing 0.1% *w*/*v* Tween^®^ 80 keeping sink conditions maintained. The test was done in a thermostatically controlled shaking water bath set at 100 rpm and 37 °C. At previously determined time intervals (1–72 h), samples were centrifuged, and the filtrate was analyzed by the previously mentioned LC-MS-MS method. The % FS and CAR released were calculated in respect to the theoretical initial drug (*n* = 3).

LNC release profiles were fitted to different mathematical kinetic models and the best fit was assessed by regression analysis of the plot. The Excel add-in, DDsolver, was used for modeling and comparison of different drug release profiles.

### In vivo studies

#### Animals

Swiss albino male mice weighing 25–30 gm were procured from the Faculty of Medicine, Alexandria University animal house. Mice were kept under 12-h light/dark cycle standard lab setting with access to water and food ad libitum. Mice were acclimatized for 7 days to the living conditions before starting the experiment. All experimental procedures complied with the ARRIVE guidelines and followed the European Parliament Directive 2010/63/EU for animal experiments. Experimental protocol was approved by the Institutional Animal Care and Ethics Committee of the Faculty of Pharmacy, Alexandria University, Egypt (approval code: AU-06-2023/4/12 − 2/ 162).

#### In vivo tracking of brain accumulation

Eighteen male Swiss albino mice were randomly divided into 3 groups. In vivo distribution of intraperitoneal (IP) injected Cou-6 solution and fluorescently labeled Cou-6@LNC and MAN-Cou-6@LNC was monitored over 24 h using an in vivo imaging system (IVIS, In vivo photon imager, Biospace lab, France). At 0.5, 1, 2, 3, 5, 24 h following injection, mice were anesthetized for fluorescence imaging at excitation and emission wavelengths of 457 and 501 nm, respectively. Fluorescence imaging of the brains following excision was also performed at the 5 h interval. The fluorescence intensity was measured by the IVIS software.

#### Kindling induction, assessment and treatment protocol

A 30-day chronic study was conducted by a total of 15 sub convulsant IP doses of PTZ (35 mg/kg) administered every other day. Mice were monitored for 30 min following PTZ administration to assess seizure intensity according to modified Racine score (Table 1) [42]. Mice were considered to be fully kindled if stage 5 of seizures is displayed in two successive doses of PTZ, i.e., clonic-tonic seizures [43, 44].

**Fig. 1:**
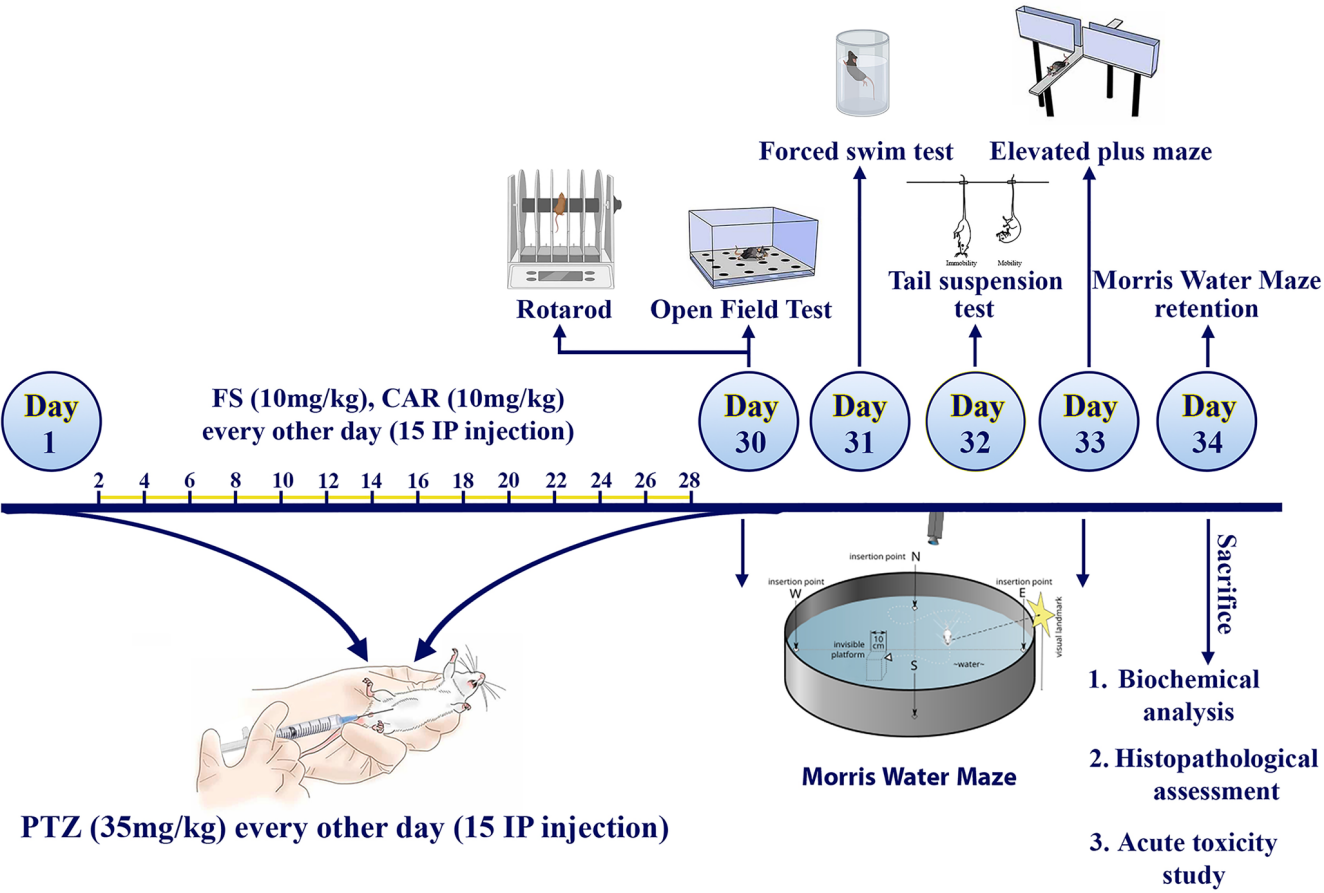
Experimental design and treatment schedule for the evaluation of the antiepileptic effects of FS, CAR, FS/CAR, FS/CAR@LNC and MAN-FS/CAR@LNC

A total of 56 Swiss albino mice were subdivided randomly into seven groups with eight mice each. Group 1 (healthy group); received normal saline 0.9% daily and group 2 (control group) received PTZ every other day. The other 5 groups received FS, CAR, FS/CAR, FS/CAR@LNC or MAN-FS/CAR@LNC alternating with PTZ equivalent to10 mg/kg FS or CAR. Behavioral assessment was carried on at different days at the end of the study as mentioned in (Fig. 1). By the end of the study, mice scarification by cervical dislocation was done. Brains were excised, washed with saline and divided into two equal halves for additional biochemical analysis and histopathological evaluation.

**Table 1:** Racine scoring for seizures assessment

Racine score	Behavioral expression
0	No changes in animal behavior
1	Facial and ear twitches
2	Myoclonic jerks not accompanied with rears
3	Myoclonic jerks accompanied with rears
4	Falling down into side position, tonic-clonic seizures
5	overfalling down into back position, generalized tonic-clonic seizures

#### Behavioral assessment

##### Rotarod test

The rotarod was handled to monitor motor coordination as a measure of neurotoxicity. The rotarod apparatus consisted of a non-slippery roller segmented into 3 segments to allow three mice walking at the same time. All mice practiced a 3-day training schedule to investigate a steady performance baseline [45]. Mice were trained at 3 different accelerations (10,15 and 25 rpm). After training, mice were tested on the highest speed and latency to fall off was recorded and calculated as mean of three trials. The apparatus was cleansed with 70% ethanol before each trial.

##### Open field test

The open field test is a prevailing assessment of anxiety, general motor activity and exploratory behavior. The open field equipment is an open wooden arena (31 × 31 × 30 cm) with a floor divided into 25 equal squares and placed in a dark room with moderate light. Whereas normal mice seek protection by moving at the box periphery rather than the center, less anxious mice are supposed to spend more time in the center. Each mouse was solely placed in the middle of the equipment then monitoredfor 5 min. Locomotor activity was assessed by counting the total number of crossings and that of rears [46], also investigating whether mice prefer staying in the center or at the periphery of the equipment [47]. 70% ethanol solution was used for wiping the equipment after each trial.

##### Elevated plus maze

The elevated-plus maze model was formed from a wooden arena in the form of PLUS sign constituting two opposite closed arms and two open arms of similar dimensions (30 × 8 cm) with 15-cm high black walls. The test was done in a quiet room with the apparatus elevated 45 cm above the floor. Mice were separately placed in the central platform facing one of the open arms and left for 5 min to explore the area. The degree of animal anxiety was assessed by the entries number escalation and the time spent in the open arms [44].

##### Forced swimming test

This test serves to assess the locomotory and antidepressant behaviors of mice. Both active and passive swimming movements were recorded and considered as depression index [47]. Mice were forced to swim in water tank of dimensions; 20 × 20 × 40 cm with no exit at 15 cm depth maintained at 25 ± 1 ℃. Water was replaced after each mouse.

##### Tail suspension test

Tail suspension test reveals depressive-like behavior in animals by staying immobile. The test relies on observing mice depressive behavior when placed in a stressful inescapable situation. Under these conditions, depressed mice show agitation followed by immobility. Mice were suspended by tails with the help of a thread at a height of 350 mm from the floor. The total period of immobility is recognized as loss of hope (depressive behavior) [48].

##### Morris water maze test

The Morris water maze test was done with the aim of investigating mice cognitive and hippocampal-dependent spatial learning ability as antecedently reported [35] with some modifications. The test was performed in a large metallic circular tank filled with tap water of temperature 25 ± 1 °C. The tank was hypothetically divided into four quadrants referring to the target quadrant having an elevated platform (1 cm below the surface). Briefly, mice training was done 4 times a day, repeated at 5 min interval, for 4 consecutive days. Whereas in all trials the platform position was fixed, the starting quadrant was changed in each trial. Mice were allowed one minute to find the hidden platform in each trial. On day 5, investigation of the spatial memory was done by removing the platform and allowing mice to swim freely for one minute. The time taken to locate the platform (escape latency) and the time spent in the target quadrant were calculated to measure the extent of neurodegenerative potential and memory function.

#### Neurochemical assessment

##### Preparation of brain homogenate

Homogenization of frozen brain sections stored at -80 °C was carried out in ice-cold phosphate buffer (pH 7.4, 10 mM) reaching a 10% w/v homogenate concentration. Homogenate centrifugation was done at 10 K g at 4 °C for 15 min. The separated supernatant was then used for biochemical assessment.

##### Determination of brain-derived neurotrophic factor (BDNF) level

The level of BDNF in brain homogenate was measured using Elabscience^®^ Mouse BDNF enzyme-linked immunosorbent assay (ELISA) kits according to manufacturer’s instructions (Cat. No. E-EL-M0203). Results are expressed as ng/mg protein.

##### Measurement of serotonin level

The level of serotonin (5-HT) monoamine neurotransmitter in brain tissue homogenate was precisely investigated by ELISA kit (ab133053) Abcam, UK according to the manufacturer’s instructions and expressed as ng/gm protein.

##### Determination of glutamate activity

Glutamate level was investigated by colorimetric assay method. All materials were equilibrated to room temperature prior to analysis. Reaction mixtures were prepared by mixing glutamate assay buffer and developer solution with enzyme mix VIII. These reaction mixtures were then added into wells containing either the standard or samples then incubated for 30 min at 37 °C protected from light. Glutamate level was investigated spectrophotometrically at 450 nm. Results are expressed in nmol of glutamate/g tissue considering that one 1U of enzyme activity is equivalent to 1 nmol of glutamate/g tissue.

##### Determination of pro-inflammatory markers

ELISA kits were used to estimate the level of the proinflammatory cytokines; interleukin-6 (IL-6) and interleukin-1β (IL-1β) in brain tissue homogenates of kindled mice following manufacturer’s instructions. Results are expressed as pg/ mg protein.

#### Histopathological examination

Excised brain samples were fixed in 4% paraformaldehyde for 48 h at room temperature dried and cleaned then embedded in paraffin. Sections: five-micrometer thick were placed on poly-L-lysine coated slides, deparaffinized with xylene, and rehydrated in ethanol. Hematoxylin and eosin (H&E) were used for sections staining according to the standard procedure [49]. Captures at different magnifications were taken using an Olympus digital camera UC30 and an Olympus microscope XC30 (Germany).

#### Acute toxicity

Acute toxicity was evaluated at the end of the chronic PTZ induced kindling study by assessing the effect of treatment on general animal health throughout the treatment course in addition to histopathological and biochemical analysis. Organs (liver, kidney, and spleen) were dissected following sacrifice, and immediately fixed in 10% v/v paraformaldehyde, sectioned and stained with H&E, then microscopically inspected to observe any pathological abnormalities. For biochemical assessment, blood samples were collected on the day of sacrifice then centrifuged at 3000 × g for 15 min. Serum samples were analyzed for liver functions by measuring the level of aspartate aminotransferase (AST) and alanine aminotransferase (ALT) and kidney functions by measuring creatinine and urea levels in comparison to healthy animals.

### Statistical analysis

All experiments were performed thrice (*n* = 3) with results expression is as mean ± SD. Statistical analysis was performed using the unpaired Student’s t-test and the one-way analysis of variance (ANOVA) followed by a post-hoc Tukey’s test for multiple comparisons handled by GraphPad Prism (Version 7.04, San Diego, CA, USA) setting the difference to be significant at *p* ≤ 0.05.

## Results and discussion

### Preparation and physicochemical characterization of LNC

LNC were prepared by the solvent free phase inversion method [16]. The preparation process comprises two distinct steps [50]. Initially, the emulsion constituents are combined under magnetic stirring and heated to a temperature exceeding the phase inversion temperature (PIT), resulting in the formation of a water-in-oil (W/O) emulsion. Subsequent cooling below the PIT induces a transition to an oil-in-water (O/W) emulsion. The system is then subjected to multiple thermal cycles that traverse the phase inversion zone (PIZ) [16]. At lower temperatures, a W/O emulsion exhibiting elevated electrical conductivity is formed, attributed to the surfactant monolayer possessing a large, positive spontaneous curvature. As the temperature increases, this curvature shifts to negative values, leading to the reformation of a W/O emulsion and a marked decrease in conductivity, approaching 0 mS/cm [50]. Within the PIZ, a gradual variation in conductivity is observed, indicative of the presence of bicontinuous microemulsion structures, where the spontaneous curvature nears zero. In the subsequent step, disruption of the microemulsion formed within the PIZ facilitates the generation of stable nanocapsules [50]. This transformation is induced by an irreversible shock, achieved through the sudden dilution of the emulsion with cold water at a temperature 1–3 °C above the onset temperature of O/W emulsion formation [16].

**Fig. 2:**
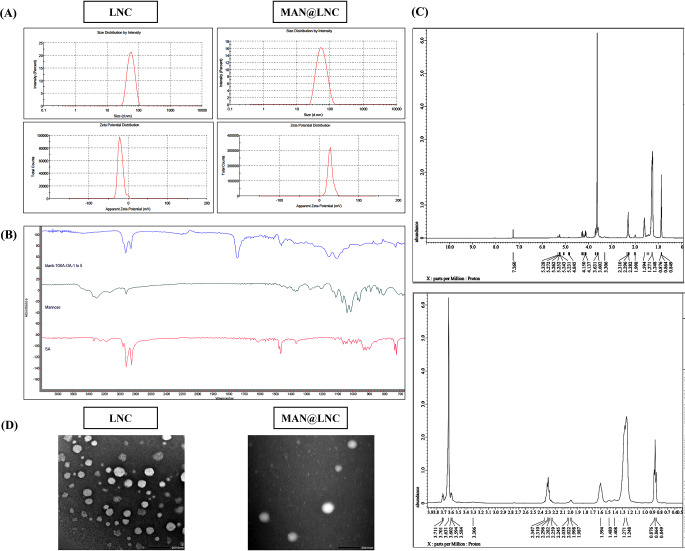
(**A**) Size distribution by intensity and zeta potential of LNC and MAN@LNC, (**B**) FTIR spectra of stearylamine (SA), mannose (MAN) and the selected MAN@LNC (F6), (**C**) ^1^H-NMR spectrum of the selected MAN@LNC (F6) with upper panel range from 0–10 and lower panel focusing on the range 0.5-4 and (**D**) TEM images of LNC and MAN@LNC × 40k. The scale bar represents 200 nm

LNC as first formulated by Heurtault et al. [16], were shown to possess high capability of encapsulating hydrophobic drugs in their oily core with sustained release over a prolonged duration [38]. In the current study, LNC were composed of equal weights of the medium chain triglyceride; Labrafac as the oily core with the pegylated surfactant; Kolliphor HS15 forming the tensioactive shell [51]. In order to reduce the heating-cooling cycle temperature range during LNC formulation, the long chain unsaturated fatty acid; oleic acid was incorporated as a fluidizer of the surfactant monolayer [37] as previously mentioned by Amara, R.O., et al. [52]. This reduction in temperature range is crucial in FS protection against degradation [36]. As shown in Table 2, LNC (F1) displayed colloidal properties comparable to those previously mentioned in the literature [22, 53] with small particle size (55 ± 0.21 nm) and narrow size distribution (0.031 ± 0.02) indicating homogenous population and confirming the reported monodispersity of LNC [53] (Fig. 2A). Regarding ZP, LNC (F1) showed a negative zeta potential of -19.6 ± 3.05 mV attributed to the presence of small fractions of surfactants after hydrolysis forming negatively charged polar groups [54]. This negative charge together with effect of bulky PEG chains of Kolliphor^®^ HS 15 which leads to steric hindrance, prevents aggregation and confers colloidal stability to LNC formulation [55].

MAN coating of LNC was based on Schiff’s base chemistry. This was accomplished by first modifying LNC via the addition of SA as a lipophilic cationic molecule presenting a positively charged primary amine group on the surface of LNC. This was then followed by the reaction of this amine group with the aldehyde group of the opened ring MAN. In order to optimize MAN functionalization, 2 concentrations of SA (1 and 2 mg/mL) were tested. Another factor controlling efficient grafting of MAN on the surface of LNC is the molar ratio of SA: mannose [40]. The molar ratios investigated were 1:5 and 1:10. Successful mannosylation was confirmed via changes in charge density, FTIR and ^1^H-NMR.

Whereas SA incorporation (F2 and F5) showed no significant change (*p* > 0.05) in particle size and PDI when compared to blank LNC (F1), ZP switched from negative (-19.6 ± 3.01 mV) to positive charge (11.5 ± 2.56 and 20.5 ± 2.5 mV for F2 and F5, respectively) as shown in Table 2. This confirms the orientation of SA primary amine groups on the surface of LNC which was also dependent on SA concentration as shown by the statistically significant difference between F2 and F5 (*p* < 0.001).

Mannosylation was tested at 2 different molar ratios of SA: mannose (1:5 and 1:10). Again, particle size was preserved with no significant difference discerned compared to the uncoated formulation (*p* > 0.05). A slight increase in PDI was observed with increasing MAN concentration but was still below 0.3 reflecting monodispersity of the coated LNC.

Mannose grafting in different molar ratios (1:5 and 1:10) showed only a slight insignificant increase in positive charge (*p* > 0.05) in case of LNC modified with 1 mg/mL SA (F3 and F4) compared to F2. For LNC with 2 mg/mL SA, both molar ratios (1:5 and 1:10) SA: mannose (F6 and F7) showed a significant increase in positive charge (26.85 ± 1.05 and 27.7 ± 0.3 mV, respectively) when compared to F5 (20.5 ± 2.5 mV). This change in surface charge reflects efficient MAN coating [56] and allows for better stability of LNC due to electrostatic repulsion forces.

Based on the change in ZP observed and the insignificant difference in charge between F6 and F7, 2 mg/mL SA and 1:5 SA: mannose molar ratio (F6) was selected as the optimum mannosylation conditions.

In all formulations tested, the change in colloidal properties following FS and CAR loading was insignificant (*p* > 0.05).

**Table 2:** Mannosylation conditions and colloidal properties of LNC formulations (*n* = 3)

Code*	SA (mg/mL)	SA: Mannose (Molar ratio)	Size (nm)	PDI	Zeta potential (mV)
F1	-	-	55.01 ± 0.21	0.03 ± 0.02	-19.6 ± 3.01
F2	1	-	54.96 ± 1.55	0.07 ± 0.01	11.5 ± 2.56
F3	1	1:5	54.94 ± 2.86	0.06 ± 0.01	13.5 ± 2.31
F4	1	1:10	64.75 ± 4.49	0.14 ± 0.04	14.6 ± 1.89
F5	2	-	52.43 ± 4.01	0.12 ± 0.03	20.5 ± 2.52
F6	2	1:5	53.17 ± 4.06	0.11 ± 0.02	26.9 ± 1.05
F7	2	1:10	54.79 ± 3.79	0.23 ± 0.03	27.7 ± 0.31

*All formulations were of similar composition regarding Kolliphor, labrafac and oleic acid

### Fourier transform infrared spectroscopy (FTIR)

FTIR spectra of MAN, SA and the selected MAN coated formulation; MAN@LNC (F6) are presented in Fig. 2B. MAN showed characteristic peaks at 3292.8 cm^− 1^ and 2916.2 cm^− 1^ for -OH and -CH_2_ stretching vibrations, respectively. Also, MAN expressed more peaks at 1035 cm^− 1^ and 1419.6 cm^− 1^ indicating C-O-C stretching of either alcohol or aldehyde groups in MAN Considering SA spectrum, -NH_2_ stretching of primary amine group appeared at 3330.6 cm^− 1^. Vibrational signals at 2914.7 cm^− 1^ and 2847.5 cm^− 1^ are more intense comparable to MAN indicating -CH_2_ and -CH_3_ stretching of the long alkyl chain in SA. Furthermore, vibrational peak at 1607.6 cm^− 1^ specific for -NH_2_. Spectrum of MAN@LNC showed lower intensity peak at 3374.7 cm^− 1^ indicating an interaction between -NH_2_ of SA and -OH stretching of MAN. A peak appeared at 1607.6 cm^− 1^ with lower intensity than that of SA confirming the conversion of primary amine (stearyl amine) into secondary amine in the coated LNC thus confirming secondary amine linkage between aldehyde group in MAN and primary amine group in SA [57].

### Proton nuclear magnetic resonance

^1^H-NMR spectrum of MAN@LNC (F6) displayed a long alkyl chain proton signal at δ 1.26 ppm and a low intensity proton signal of -NH at δ 2.29 ppm pinpointing the secondary amine linkage between SA and MAN (Fig. 2C). This small intensity signal has been taken as a confirmation of the glycosylation of MAN with long chain stearyl amine [57].

### Morphological examination

TEM imaging revealed spherical structures with homogenous size distribution for both LNC and MAN@LNC [22, 52] as demonstrated in Fig. 2D. The similarity in size observed follows the results obtained by DLS showing an insignificant difference in size following mannosylation (*p* > 0.05).

### LC-MS-MS validation method

Calibration standards of both FS and CAR were prepared in the concentration range 0.1–2 µg/mL. The analytical method was partially validated for linearity. A blank sample was used to validate the specificity of the method. The detection of the target compounds was achieved using selective reaction monitoring (SRM) of the ion molecular peak of FS (m/z: 287.138) and quantifier daughter peaks (m/z: 213.173) whereas CAR (m/z: 135.14) was detected at positive electrospray ionization mode (ESI). The molecular ion peak of CAR did not show enough stability to be used as precursor ion, however, the stable daughter fragment was initially selected and used for quantitation. Peak retention times and linearity for FS and CAR were 1.76 and 2.54, respectively (Figure S1).

### Entrapment efficiency and drug payload

For %EE determination, FS and CAR were analyzed using the LC-MS-MS method mentioned in the section “LC-MS-MS validation method”. The lipophilic nature of FS and CAR permitted their incorporation in the oily core with a %EE exceeding 99% for 1 and 2 mg/mL of the final dispersion volume initial drug loaded. This high %EE has been analogously reported for other lipophilic drugs following encapsulation into LNC [52, 55]. Drug payload for FS and CAR was ≈ 8 and 16 mg/g for 1 and 2 mg/mL initial drug loaded, respectively. No change in %EE or drug payload was observed following MAN coating (*p* > 0.05).

### Drug release

**Fig. 3:**
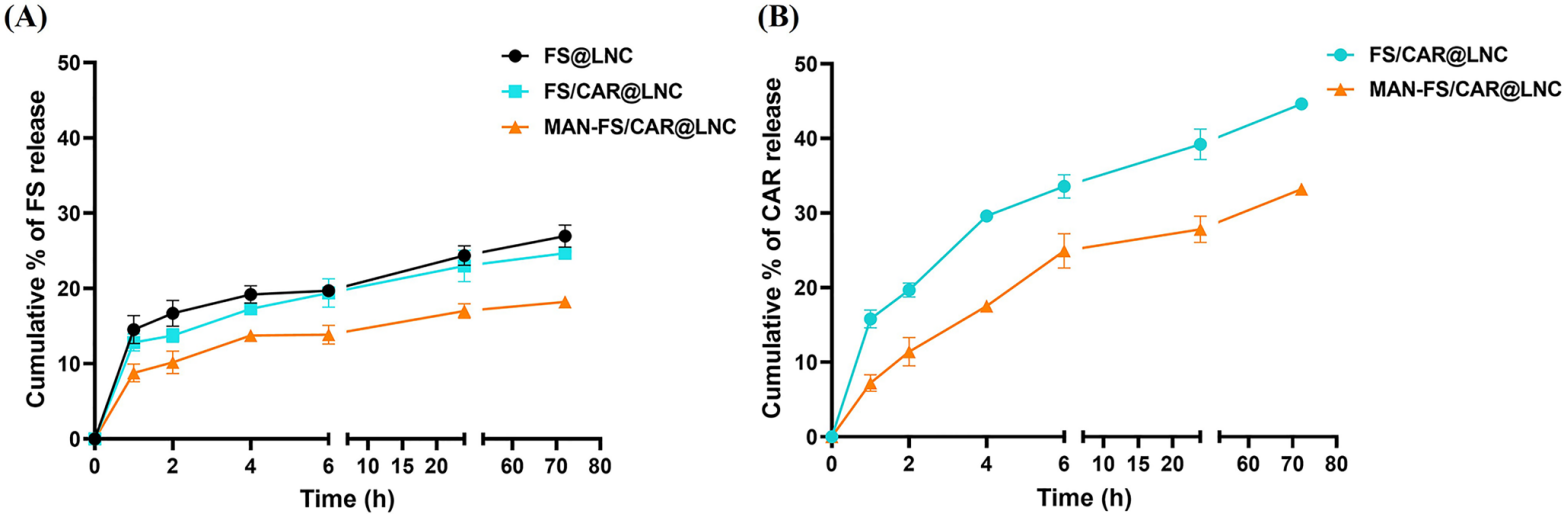
(**A**) FS and (**B**) CAR release profiles from LNC formulations over 72 h at 37 °C (*n* = 3; data are shown as mean ± SD)

The in vitro release of FS and CAR from FS@LNC, FS/CAR@LNC and MAN-FS/CAR@LNC was studied over 72 h (Fig. 3). FS@LNC showed sustained release of FS with a limited burst effect (15% after 1 h) reaching a maximum of 26.95% following 72 h. This highly sustained release profile reflects successful encapsulation of FS in the oily core of LNC and is in accordance with previous findings showing similar release patterns [37, 38]. As shown in Fig. 3A, co-encapsulation of FS and CAR did not significantly affect FS release with FS/CAR@LNC showing nearly similar FS release pattern over 72 h. On the other hand, MAN coating (MAN-FS/CAR@LNC) showed a significant reduction in FS release with only 8% released after 1 h reaching a maximum of ~ 18.22% after 72 h (*p* > 0.05). Also, CAR release from FS/CAR@LNC (Fig. 3B) was sustained (15.78 and 44.6% after 1 and 72 h, respectively). Again, a significant decrease in cumulative % CAR released (*p* < 0.05) was observed following MAN coating (MAN-FS/CAR@LNC) compared to FS/CAR@LNC. This reduction in drug release of both FS and CAR reflects successful mannosylation of LNC forming an outer coating envelop thus impeding drug release [39]. A similar pattern was previously reported for gemcitabine release from mannose coated solid lipid nanoparticles [58].

The mechanism of FS and CAR release from FS@LNC, FS/CAR@LNC and Man-FS/CAR@LNC was investigated by fitting to different release models, specifically the zero-order, first-order, Higuchi, Korsmeyer-Peppas and Hixson-Crowell models. To evaluate the best fit data, the largest correlation coefficient (r) and smallest mean standard error (MSE) were the statistical parameters utilized. Results revealed that FS and CAR exhibited diffusion-controlled release from FS@LNC, FS/CAR@LNC and MAN-FS/CAR@LNC depending on maximum r and minimum MSE values observed for Korsmeyer-Peppas model (Table S1). Furthermore, the release exponent value (n) ≤ 0.5 was determined as being Fickian diffusion. Previous reports on the kinetics of controlled lipophilic drug release from LNC showed a 2-step process, firstly diffusion of solubilized drug from the oily core then partitioning to the surrounding aqueous release medium [59].

### In vivo tracking of brain accumulation

**Fig. 4:**
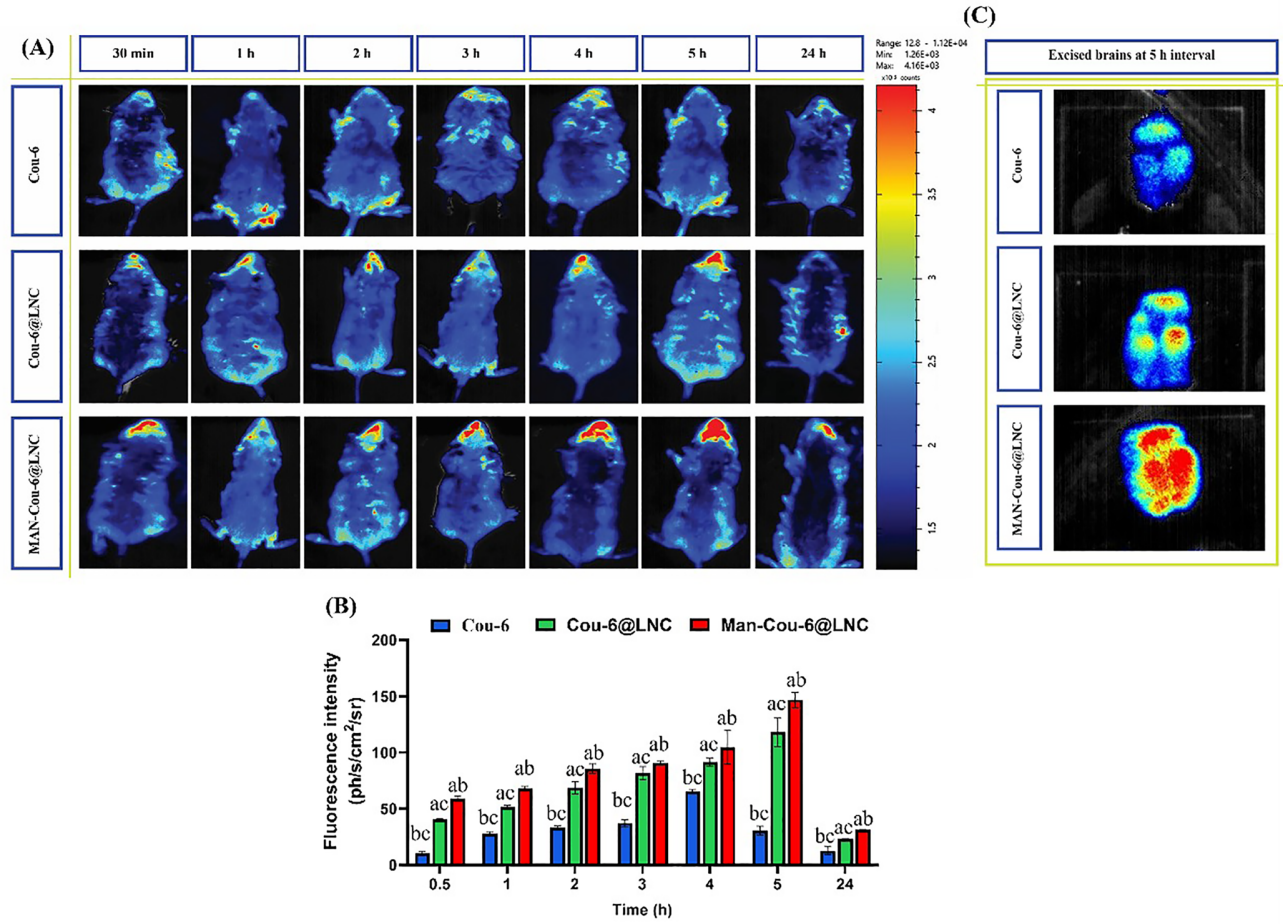
(**A**) In vivo fluorescent imaging of mice following IP administration of Cou-6, and MAN-Cou-6@LNC at different time points (0.5, 1, 2, 3, 4, 5, 24 h), (**B**) Quantitative evaluation of fluorescence signal intensity at different time intervals (Cou-6@LNC *n* = 3; data are shown as mean ± SD) and (**C**) Ex vivo fluorescent imaging of excised mice brains at 5 h interval. *Data presented as mean ± standard deviation (SD) (n = 3). (*^*a*^*p≤0.05 vs. Cou-6*, ^*b*^*p ≤ 0.05 vs. Cou-6@LNC*, ^*c*^*p ≤ 0.05 vs. MAN-Cou-6@LNC*

Improving receptor targeting and BBB penetration with specific drug accumulation in the brain tissues is a challenge facing seizures treatments. In the current work, the effect of nanoencapsulation and MAN BBB targeting via ligand/receptor strategy on brain accumulation of Cou-6@LNC and MAN-Cou-6@LNC was tracked over 24 h using IVIS (Fig. 4A). Cou-6 was used as a control. The mean fluorescence intensity at each time point was also measured (Fig. 4B). The results showed a stronger fluorescence signal for MAN-Cou-6@LNC compared to Cou-6 and Cou-6@LNC at all time points reaching maximum fluorescence intensity at 5 h. Excised brains after 5 h are shown in Fig. 4C. These results provide riveting evidence of remarkable BBB penetration and brain targetability via glucose transporters overexpressed on the BBB thus enhancing uptake and brain accumulation. These results come in accordance with previously formulated human serum albumin nanoparticles [23] and oral brain targeting nanoparticles [60] where mannosylation resulted in enhanced cellular uptake and brain targetability.

### In vivo efficacy study

#### Effect of treatment on seizure severity score in PTZ induced kindling in mice

**Fig. 5:**
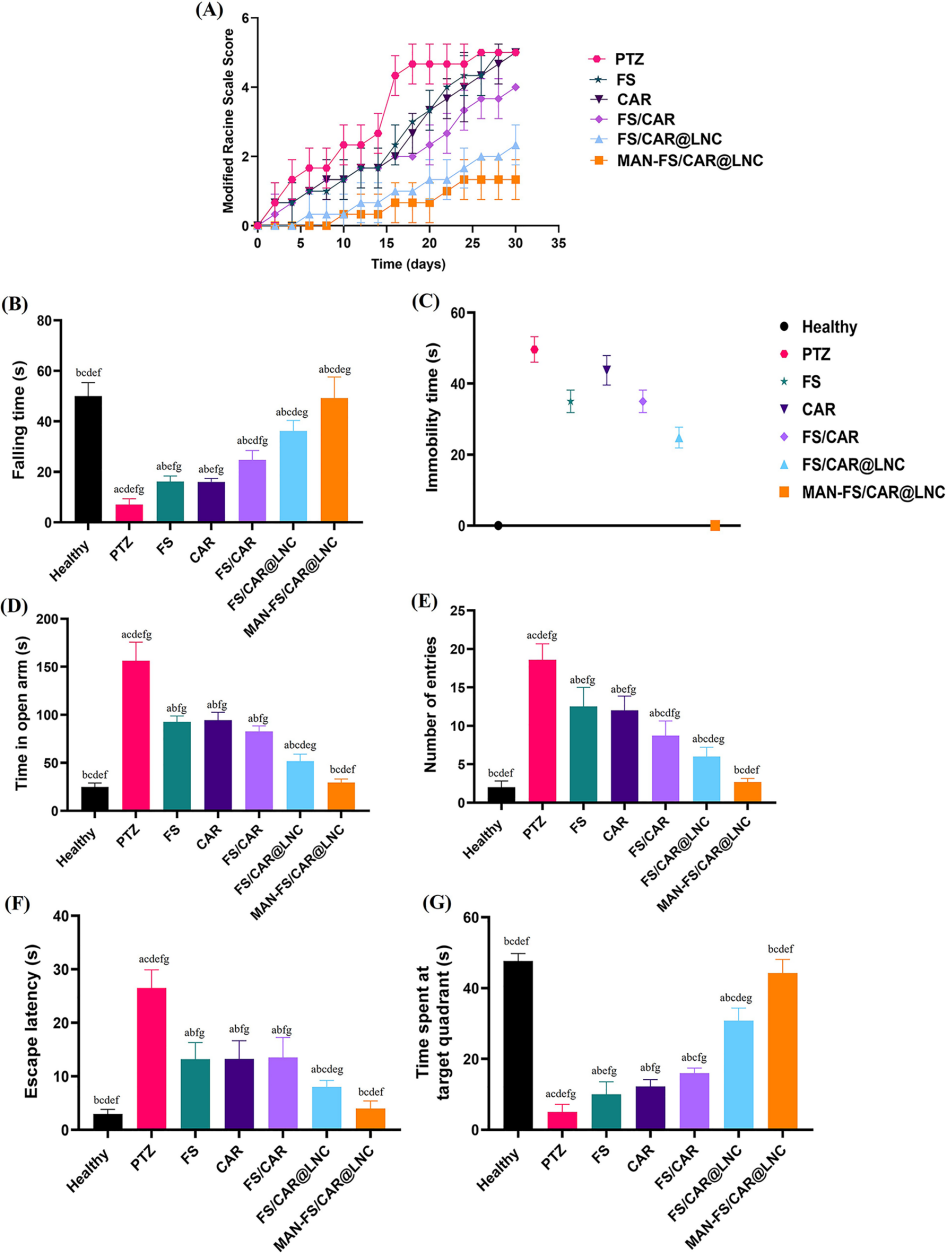
Effect of FS, CAR, FS/CAR, FS/CAR@LNC and MAN-FS/CAR@LNC treatment on the in vivo behavioral assessment of mice in PTZ-induced chronic epilepsy model: (**A**) Modified Racine Score scale, (**B**) Rotarod test falling time (s), (**C**) Immobility time in tail suspension test (s), (**D** and **E**) time spent in open arm (s) (**D**) and number of entries to the open arm (**E**) in the elevated plus maze test and (**F** and **G**) Escape latency (**F**) and time spent at target quadrant (**G**) in Morris water maze test. *Data presented as mean ± standard deviation (SD) (n = 8). (*^*a*^*p≤0.05 vs. healthy control*, ^*b*^*p ≤ 0.05 vs. positive control (PTZ)*, ^*c*^*p ≤ 0.05 vs. FS*, ^*d*^*p≤0.05 vs. CAR*, ^*e*^*p≤0.05 vs. FS/CAR*, ^*f*^*p≤0.05 vs. FS/CAR@LNC and*^*g*^*p≤0.05 vs. MAN-FS/CAR@LNC)*

PTZ, GABA-A receptor antagonist, is utilized in chronic epilepsy model induction by altering GABA and glutamate levels [61]. Chronic PTZ sub-convulsive doses are the root of excessive neurodegeneration and excitotoxicity thus disturbing the central role of amygdala in emotions and emotional behavior [62]. In the current study, protracted treatment with a sub convulsive PTZ dose (35 mg/kg, IP) significantly (*p* < 0.05) increased seizure severity score in comparison to the healthy group. PTZ control group showed the highest Racine score of 5 starting from day 16 and exhibited generalized tonic-clonic seizures (Fig. 5A). Treatment with FS or CAR showed slight improvement in seizure severity with the highest Racine score recorded on the 28th day of treatment. This could be attributed to the low dose of FS and CAR used (10 mg/kg) compared to previous studies [31, 35]. On the other hand, when FS and CAR were combined, a synergistic effect was clear reaching the 4th stage in the Racine score. The anticonvulsant effect is attributed to the modulating effect of FS on GABAergic pathways through boosting GABA levels in the brain conferring [63]. Also, CAR augments the endogenous Nrf2 antioxidant pathway aiding in seizures reversal [35]. Furthermore, treatment with FS/CAR@LNC and MAN-FS/CAR@LNC significantly (*p* < 0.05) improved seizure severity when compared to FS/CAR dispersion. MAN-FS/CAR@LNC treated group showed the least Racine score among all other groups. MAN-FS/CAR@LNC consolidate the benefits of dual therapy using FS/CAR combination, LNC encapsulation which facilitates BBB permeation and finally MAN coating which enhances BBB permeation through glucose transporter-1 (GLUT-1) receptors overexpressed on BBB [25].

#### Effect on locomotor activity (rotarod test)

Rotarod was used to monitor mice motor function. Results are shown in Fig. 5B. PTZ-group demonstrated a significant (*p* < 0.001) decrease in latency time before falling from rotarod apparatus compared to the healthy group (7 ± 2.39 vs. 50 ± 5.35 s, respectively). This confirmed the capability of PTZ to induce motor dysfunction as previously reported [44]. Groups receiving FS or CAR showed quite significant prolongation in time to fall compared to the PTZ- group (*p* ≤ 0.05) which was further increased by the FS/CAR combination (16.2 ± 2.17, 16 ± 1.41 and 24.75 ± 3.73 s for FS, CAR and FS/CAR, respectively). This could be attributed to the synergistic mechanisms of FS which restores dopaminergic neurons [64] and CAR which plays a crucial protective role in the inflammatory cascade thus protecting cholinergic neurons and restoring motor function [65]. FS/CAR encapsulation into LNC (FS/CAR@LNC) significantly increased time to fall (36.25 ± 4.15 s) compared to unloaded drugs or the PTZ-group (*p* < 0.05). Furthermore, MAN-FS/CAR@LNC restored the normal locomotor function with insignificant statistical difference (*p* > 0.05) comparable to the healthy group (49.17 ± 8.37 s) reflecting successful targeting to the BBB.

#### Effect on anxiety-like behavior

Epilepsy is commonly accompanied by anxiety which occurs either preictal (in advance to seizure), ictal (as part of the seizure signs and symptoms), and postictal (within 72 h of a seizure) via a bidirectional relationship [66]. Epilepsy is accompanied by dysregulation in the hypothalamic pituitary adrenocortical system together with the imbalance between the excitatory glutamatergic system and the BDNF system. This bizarreness in neuro-transmittance involved in the pathogenesis of various mental illnesses including anxiety and depression [66].

##### Open field test

Behavioral parameters to assess the exploratory behavior and anxiety of mice were recorded (Table 3). The PTZ-group exhibited a highly significant difference (*p* ≤ 0.05) in both; latency to move or rear along with a reduced number of crossed squares or rears in comparison to the healthy control. This reflects marked impairment of locomotion and anxiety-like behavior. Administration of FS or CAR either as a single treatment or in combination showed a significant (*p* ≤ 0.05) decline in latency to move or rear with a significant (*p* ≤ 0.05) increase in the number of crosses compared to the PTZ-group. This improvement was mostly pronounced in the FS/CAR group. The increase in the number of rears compared to PTZ reached significance (*p* ≤ 0.05) only in case of FS/CAR treated group. Although free drugs accomplished significant improvement (*p* ≤ 0.05) compared to the PTZ-group, the improvement was not enough to restore the animal behavior to normality (Table 3). Further improvement was achieved by encapsulation in LNC; FS/CAR@LNC exhibited an insignificant difference compared to the healthy group (*p* > 0.05) in terms of latency to move or rear and number of rears, but the number of crossings was still significantly different (90.43 ± 8.14 and 145.88 ± 6.68 crossings for FS/CAR@LNC and healthy group, respectively) (*p* ≤ 0.05). Animal rehabilitation to normalcy was achieved following treatment with MAN-FS/CAR@LNC demonstrating results comparable to the healthy group with no significant difference (*p* > 0.05) in all the parameters assessed.

**Table 3:** Effect of different treatments on animal behavior in open field and forced swim tests in a chronic epilepsy mouse model (*n* = 8; data are shown as mean ± SD)

	Healthy	PTZ	FS	CAR	FS/CAR	FS/CAR@LNC	MAN-FS/CAR@LNC
*Open field test*							
Latency to move (s)	0	170 ± 11.2	12 ± 3.3	85 ± 11.2	5.6 ± 3.3	0	0
No of crossing	145.9 ± 6.7	44 ± 4.2	56.8 ± 3.7	58.75 ± 1.3	67.6 ± 6.5	90.4 ± 8.1	145.88 ± 6.7
Latency to rear (s)	31.3 ± 5.4	187.7 ± 7.3	156.5 ± 5.6	136.8 ± 6.7	88 ± 7.9	40.8 ± 5.3	33 ± 4.1
No of rears	25.8 ± 3.8	6.1 ± 1.9	7 ± 2.7	7.8 ± 1.9	11.3 ± 1.3	25 ± 4.5	30 ± 5.7
*Forced swim test*							
Swimming time (s)	58.33 ± 2.36	48 ± 2.5	46.7 ± 4.7	47.5 ± 2.5	49.5 ± 2.9	52.5 ± 3.54	57.5 ± 3.8
Immobility time (s)	1.25 ± 2.17	12 ± 2.5	13.3 ± 4.7	12.5 ± 2.5	10.8 ± 0.8	8.6 ± 2.3	4 ± 1.4

Studies were conducted on male Swiss albino mice (*n* = 8). Values were reported as means ± SD

##### Elevated plus maze

PTZ-kindled group showed a significant increase in both the number of entries into the open arm and the time spent thererelative to the healthy group (18 ± 2.06 and 156.17 ± 19.48 s, respectively) (*p* ≤ 0.05) confirming anxiety-like behavior in mice as mentioned in Fig. 5D and E. Groups receiving either FS, CAR or FS/CAR exhibited reversal effect to the anxiety-like behavior of the PTZ-group with a significant decrease (*p* ≤ 0.05) in both entries number and time spent in open arms compared to the PTZ group. FS/CAR@LNC resulted in further improvement, but the difference was still significant compared to the healthy control (*p* ≤ 0.05). However, MAN-FS/CAR@LNC achieved the most pronounced behavior rectification with no significant difference (*p* > 0.05) in contrast with the healthy group (2.67 ± 0.47 and 2 ± 0.82 entries and 29.5 ± 3.64 and 25 ± 4.08 s spent in the open arm for MAN-FS/CAR@LNC and healthy control, respectively) indicating reversal of PTZ induced anxiety.

#### Effect on depressive behavior

The most common epilepsy consequence is the depressive episodes resulting from the multifactorial stress conferred by seizures [66]. The key evidence triggering comorbid epilepsy and depression includes microglial activation, impaired neurogenesis, neuroinflammation and neurodegeneration. Also, alteration in the neurotransmitter systems (serotonergic, monoaminergic, glutamatergic, and GABAergic) is a common cause. In addition, there is a persistent disturbance of hypothalamic pituitary adrenocortical system either by boosting activation or by subsequent depletion [7].

##### Forced swim test

The forced swim test covers the observation of both swimming and immobility time reflecting depressive status in epileptic mice (Table 3). PTZ-induced seizures impairs the locomotor function and prompts depression in mice [61]. The healthy group showed an elongated swimming period (58.33 ± 2.36 s) with only 1.25 ± 2.17 s immobility time. On the other hand, PTZ-treated mice kept swimming for 48 ± 2.45 s and were immobile for 12 ± 2.45 s reflecting behavioral despair. Treatment with FS, CAR or FS/CAR did not bring about any significant improvement (*p* > 0.05) compared to PTZ group. This depressive behavior was reversed in groups receiving FS/CAR@LNC or MAN-FS/CAR@LNC showing swimming times of 52.5 ± 3.54 and 57.5 ± 3.82 s, respectively.

##### Tail suspension test

The PTZ regimen significantly (*p* ≤ 0.05) increased immobility time (49.63 ± 3.60 s) depicting depressive behavior in mice compared to the healthy group (Fig. 5C). Treatment with FS, CAR or FS/CAR resulted in a slight decrease in immobility time and depressive-like behavior. However, FS/CAR@LNC and MAN-FS/CAR@LNC significantly reduced immobility time and reversed PTZ induced depression with MAN-FS/CAR@LNC showing a behavior comparable to the healthy group.

#### Effect on memory performance (morris water maze test)

Whereas the escape latency diminished in the healthy group together with an obvious increase in time spent in platform quadrant (3 ± 0.82 and 47.67 ± 2.05 s, respectively), spatial learning dropped with longer escape latency time and a drop-in time spent at the hidden platform in PTZ injected group (26.5 ± 3.4 and 5 ± 2.16 s, respectively) implying severe memory deficits. Spatial learning process was significantly improved by FS, CAR and FS/CAR administration (*p* ≤ 0.05) (Fig. 5F and G). Previous reports on CAR showed its role in improving memory deficits [35, 67]. Also, FS was reported to be responsible for the activation of signaling pathways in the hippocampal region leading to increment in long term memory in mice [63] in addition to amelioration of cognitive functions [68]. Encapsulation of FS/CAR in LNC resulted in further improvement with MAN-FS/CAR@LNC showing insignificant difference (*p* > 0.05) in the escape latency time and time spent in the platform quadrant compared to the healthy group (4 ± 1.41 and 44.2 ± 3.87 s, respectively).

#### Biochemical analysis

##### Determination of brain-derived neurotrophic factor (BDNF) levels

**Fig. 6:**
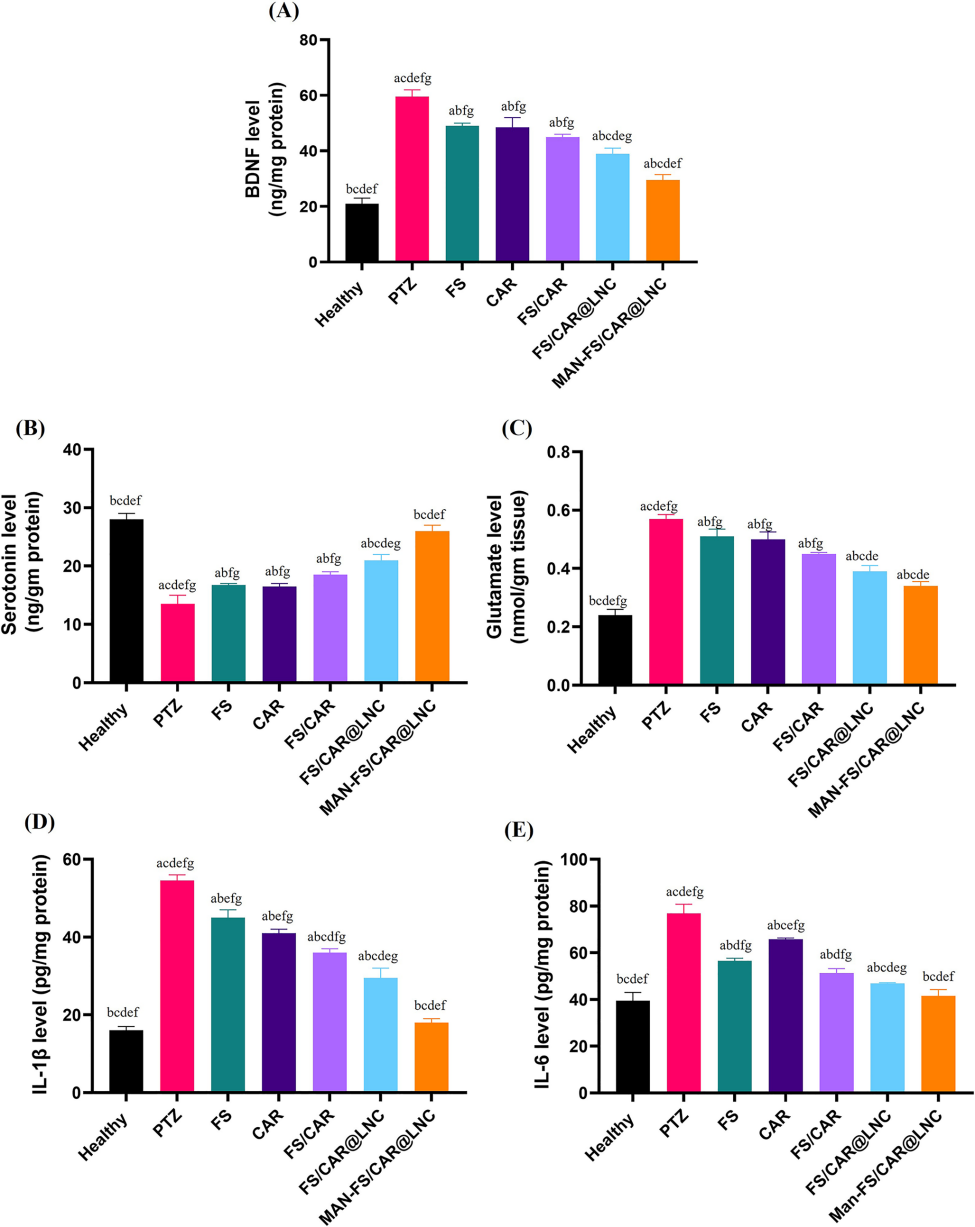
Biomarkers analysis in excised brains of different study groups (FS, CAR, FS/CAR, FS/CAR@LNC and MAN-FS/CAR@LNC): (**A**) Brain derived neurotrophic factor (BDNF), (**B**) Serotonin, (**C**) Glutamate, (**D**) IL-1β and (**E**) IL-6 protein level quantified by ELISA. *Data presented as mean ± standard deviation (SD). (*^*a*^*p≤0.05 vs. healthy control*, ^*b*^*p ≤ 0.05 vs. positive control (PTZ)*, ^*c*^*p ≤ 0.05 vs. FS*, ^*d*^*p≤0.05 vs. CAR*, ^*e*^*p≤0.05 vs. FS/CAR*, ^*f*^*p≤0.05 vs. FS/CAR@LNC and*^*g*^*p≤0.05 vs. MAN-FS/CAR@LNC)*

Normally the neurotrophin BDNF is incorporated in neuronal survival and differentiation. Epileptogenesis is accompanied by aberrant increment in neurogenesis and dendrites remodeling resulting from excessive augmentation of BDNF in specific areas in the hippocampus [66] revealing an excitatory role on both animal brain and cultured neurons [7]. BDNF involvement in the functioning of essential neurons in epilepsy [69] and depression [70] comorbidities was previously reported. Moreover, glutamatergic system regulation is one of the primary responsibilities of BDNF system. Figure 6A. shows BDNF level in brain tissue homogenate of mice following treatment regimen. FS and CAR showed a comparable decrease in BDNF level of 17.6% relative to PTZ group (*p* ≤ 0.05) which was further augmented to 24.6% by FS/CAR combination. FS and CAR were previously reported to modulate the expression of BDNF restoring oxidation and neuroinflammation [66, 71]. The combination of both FS and CAR thus augmented the neuropsychiatric behavior and seizures control in mice. FS/CAR@LNC and MAN-FS/CAR@LNC expressed further decrement in BDNF levels compared to PTZ group (34.4 and 50.4%, respectively) (*p* ≤ 0.05) with a significant difference between the 2 groups highlighting the brain targeting capability of the MAN coated formulation.

##### Determination of serotonin level

Anxiety and depression, the signs of psychiatric complications in epileptic mice, are accompanied by neuronal excitability and switching in essential monoamine levels like dopamine, norepinephrine and serotonin [72]. In agreement with the behavioral tests findings which showed anxiety-like and depressive behavior in the PTZ group, a 2-fold reduction in serotonin level was observed compared to healthy group (*p* ≤ 0.05) (Fig. 6B). Mice receiving free drugs (FS, CAR, FS/CAR) expressed comparable significant (*p* ≤ 0.05) increase in serotonin relative to PTZ group (≈ 1.3-fold). FS was shown to inhibit monoamine oxidase activity stopping the deterioration of neurotransmitters thus boosting serotonin and noradrenaline in the frontal cortex and hippocampus [73] conferring anti-depressant effects. Also, *Nrf2* pathway activation by CAR results in modulation of the levels of monoamine neurotransmitters as serotonin [74]. This combination of FS and CAR merges the merits of both drugs demonstrating antidepressive like action in epileptic mice. FS/CAR@LNC showed a further significant increase compared to FS/CAR (21 ± 1 and 18.5 ± 0.5 ng/g protein, respectively) (*p* ≤ 0.05). Remarkably, MAN-FS/CAR@LNC achieved the highest escalation in serotonin level among other treated groups with an insignificant difference compared to the healthy group (*p* > 0.05).

##### Determination of L-glutamate activity

Glutamatergic system is considered as the focal excitatory neurotransmission system in the temporal lobe [66]exerting a pivotal role in both epilepsy and depression through its overactivation in the hippocampus [66]. Abnormal signals result in glutamate receptors damage together with oxidative stress and excitotoxicity induction [75]. In PTZ induced chronic epilepsy model, repeated PTZ injections induce a marked elevation in glutamate level [76]. Herein, a 2.4-fold increase in glutamate level in PTZ-group compared to the healthy group was recorded (*p* ≤ 0.05) (Fig. 6C). Treatment with FS, CAR and FS/CAR showed a slight but significant decrease in glutamate level related to the PTZ-group (*p* ≤ 0.05). This is in accordance with previous reports on FS as a promising neuroprotective compound controlling glutamate level [77]. Also, CAR was shown to act as an Nrf2 pathway activator which in turn reduces the level of glutamate neurotransmitter [74]. Again, this reduction in glutamate level was more pronounced following LNC encapsulation with 1.69-Fold decrease in glutamate following treatment with MAN-FS/CAR@LNC.

##### Determination of pro-inflammatory markers

Epileptogenesis is accompanied by elevation in the level of pro-inflammatory cytokines [78] leading to brain hyperexcitability via synaptic transmission enhancement. In the current study, sub convulsive PTZ injections showed a pronounced increase in pro-inflammatory cytokines production in the hippocampal tissue as consistent with earlier reports [79]. IL-1ꞵ and IL-6 were picked to explicate the effect of FS/CAR therapy either free or co-encapsulated in LNC on the inflammatory response in PTZ induced seizures (Figure D and E). PTZ group showed a 2-fold increase in IL-6 and a 4-fold increase in IL-1ꞵ when compared to the healthy group. Treatment with FS, CAR, FS/CAR resulted in a significant decrease in proinflammatory cytokines relative to the PTZ group (*p* ≤ 0.05) with FS/CAR possessing the highest anti-inflammatory effect. This amplified anti-inflammatory action is attributed to the combined anti-inflammatory action of FS via inhibition of NF-kB and COX-2 expression in the hippocampus and cortex [80] with a reduction in the overexpression of IL-6, IL-1ꞵ and TNF-α and CAR which activates Nrf2 pathway thus controlling both neuroinflammation and oxidative stress [35]. FS/CAR encapsulation into LNC resulted in further significant decrease in inflammatory markers (*p* ≤ 0.05) with the brain targeted MAN-FS/CAR@LNC restoring proinflammatory cytokines to a level comparable to the healthy group (*p* > 0.05).

Altogether, the previously discussed behavioral assessment and biochemical analysis, affirms the antecedently reported antiepileptic effect of both FS [81] and CAR [35] with modulation of depressive and anxiety-like actions. FS/CAR combination inferred an augmented synergistic effect compared to single treatments. Furthermore, FS/CAR loading in LNC enhanced epileptic seizure control through brain accumulation owing to its prolonged circulation time and suppression of the multi drug resistance transporter; P-gp efflux pump [20] overexpressed on diseased BBB [21]. Additionally, LNC were shown to confer brain permeation via different endocytic pathways of neuronal cells resulting in increased drug-brain bioavailability [17]. Increased drug accumulation via LNC encapsulation has been described for apocynin/lavender nanocapsules compared to free drug as indicated by LogBB value [22]. Successful size dependent internalization in human malignant glioma cells (U373MG cell line) was also reported [18]. Compellingly, in the current study the passive brain-targeting ability of LNC was combined with an active targeting strategy via surface coating with MAN. Throughout the in vivo study, MAN-FS/CAR@LNC was able to restore mice behavioral and biochemical parameters to normal. This confirms MAN coating capability to target the brain via GLUT-1 enhancing brain accumulation and hence epileptic seizures control. Efficient brain targeting capacity by MAN coating has been previously proved for mannosylated liposomes [26] and PLGA nanoparticles [60] in Alzheimer’s treatment.

#### Histopathological study

**Fig. 7:**
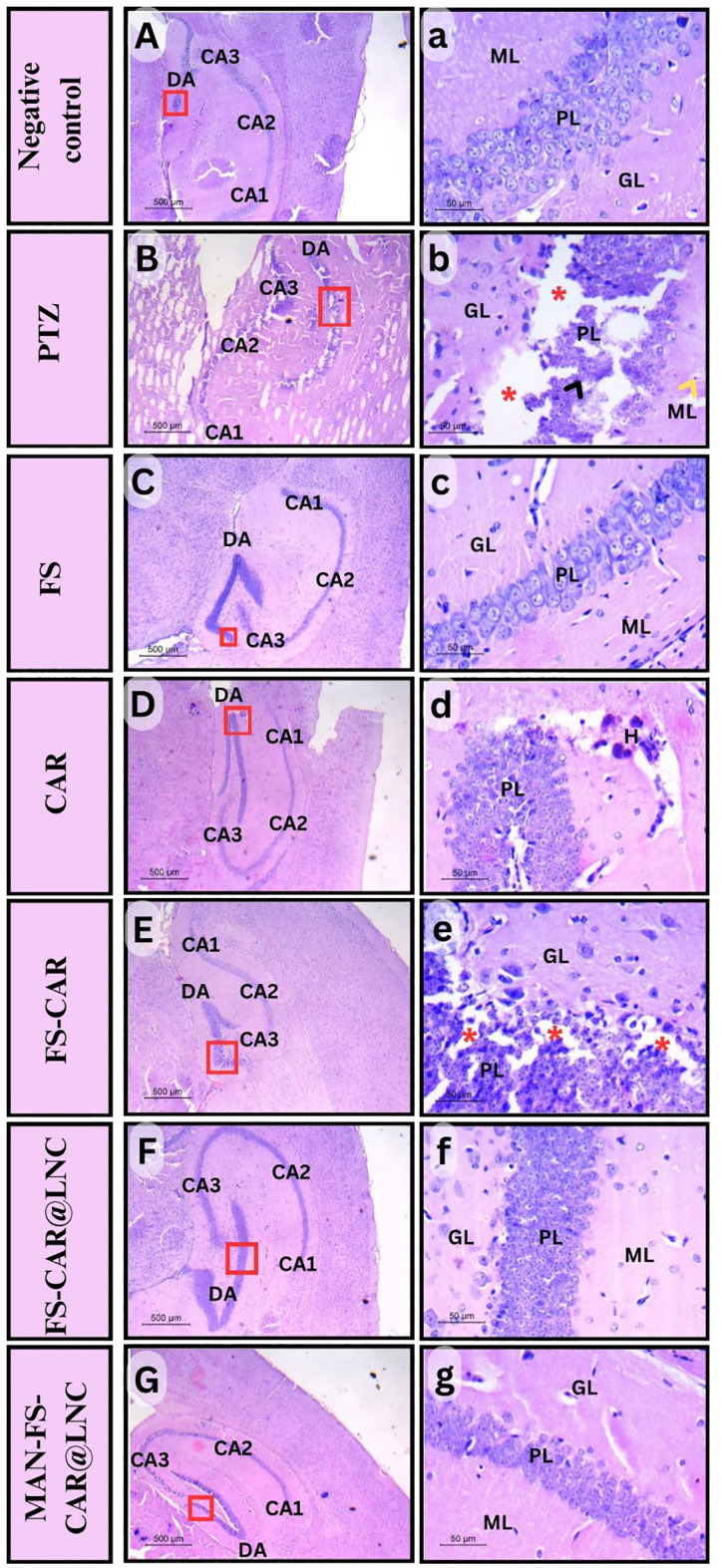
Representative H&E-stained brain samples of male rats, showing the hippocamus: Negative control group: **(A)** shows normal layer thickness and structure of hippocampus including cornu ammonis 1 (CA1), cornu ammonis 2 (CA2), cornu ammonis 3 (CA3), and dentate gyrus (DG), **(a)** Dentate gyrus (DA) consists of normally structured layers: granular layer (GL), polymorphic layer (PL), and molecular layer (ML). **PTZ group: (B)** shows degenerated, unorganized layers of hippocampus including cornu ammonis 1 (CA1), cornu ammonis 2 (CA2), cornu ammonis 3 (CA3), and dentate gyrus (DG), **(b)** Dentate gyrus (DA) reveals a degenerated granular layer (GL), vacuolations (red asterisks) in the polymorphic layer (PL), molecular layer (ML), degenerated neurons (black head arrow), and gliosis (yellow head arrow). **FS group: (C)** shows hippocampus layers including cornu ammonis 1 (CA1), cornu ammonis 2 (CA2), cornu ammonis 3 (CA3), and thickened dentate gyrus (DG). **(c)** dentate gyrus (DA) consists of a normally structured granular layer (GL), a hugely thickened polymorphic layer (PL), and a molecular layer (ML). **CAR group: (D)** showed normal hippocampus layers, including cornu ammonis 1 (CA1), cornu ammonis 2 (CA2), cornu ammonis 3 (CA3), and dentate gyrus (DG), **(d)** dentate gyrus (DA) consists of a normally structured granular layer (GL), a polymorphic layer (PL), a molecular layer (ML), and some signs of hemorrhage (H). **FS/CAR group: (E)** shows normal layer thickness and structure of hippocampus including cornu ammonis 1 (CA1), cornu ammonis 2 (CA2), cornu ammonis 3 (CA3), and dentate gyrus (DG), **(e)** dentate gyrus (DA) consists of normally structured layers: the granular layer (GL), tiny vaculations (red astriesks) in the polymorphic layer (PL), and the molecular layer (ML). **FS/CAR@LNC group: (F)** shows a normal layer of hippocampus including cornu ammonis 1 (CA1), cornu ammonis 2 (CA2), cornu ammonis 3 (CA3), and thickened dentate gyrus (DG), **(f)** dentate gyrus (DA) consists of normally structured layers: granular layer (GL), thickened polymorphic layer (PL), and molecular layer (ML). **MAN-FS/CAR@LNC group: (G)** shows normal layer thickness and structure of hippocampus including cornu ammonis 1 (CA1), cornu ammonis 2 (CA2), cornu ammonis 3 (CA3), and dentate gyrus (DG), **(g)** The Dentate gyrus (DA) consists of normally structured layers: granular layer (GL), polymorphic layer (PL), and molecular layer (ML). **Right panels (magnification X100**,** scale bar 50 μm) are higher magnifications of the insets of the corresponding left panels (magnification X 50**,** scale bar 500 μm)**

Histopathological examination focusing on hippocampal changes accompanying epilepsy for evaluation of potential protective effects of FS, CAR, FS/CAR, FS/CAR@LNC and MAN-FS/CAR@LNC was done (Fig. 7). The negative control group showed a semilunar form of dentate gyrus (DG) pointed towards the cornu Ammonis (CA). CA is generally classified into three regions: CA1, CA2, and CA3, which are distributed regularly based on the proportion of neurons. Also, the hippocampus layers: molecular (ML), granular (GL), polymorphic (PL), or hilus appear in their typical condition (Fig. 7A-a). On the other hand, PTZ group exhibited various changes in hippocampal region organization. Extensive degeneration was seen throughout the specimen, as was complete degeneration in the polymorphic layer, which included apoptotic neurons and gliosis (Fig. 7B-b).

FS and CAR treated groups revealed varying ratios of healing in various contexts. FS group displayed typical DG layers but were thicker than normal. For the CAR treated group, although all of the DG layers were of normal thickness, obvious hemorrhage was seen (Fig. 7C-D). FS/CAR combination showed an improved overall structure of the hippocampus however, the PL layer degradation still persist (Fig. 7E-e). Encapsulation of FS/CAR into LNC (FS/CAR@LNC) succeeded in restoring the normal structure of the hippocampal region without any evidence of vacuolation or degeneration (Fig. 7F-f). However, it indicated an increase in PL thickness which may enhance the input signals received from this region. Interestingly, the brain targeted formulation (MAN-FS/CAR@LNC) treated group showed rehabilitated normal hippocampal cytoarchitecture including all layer components (Fig. 7G-g) which further supports the behavioral and biochemical results obtained.

### In vivo toxicity study

**Fig. 8:**
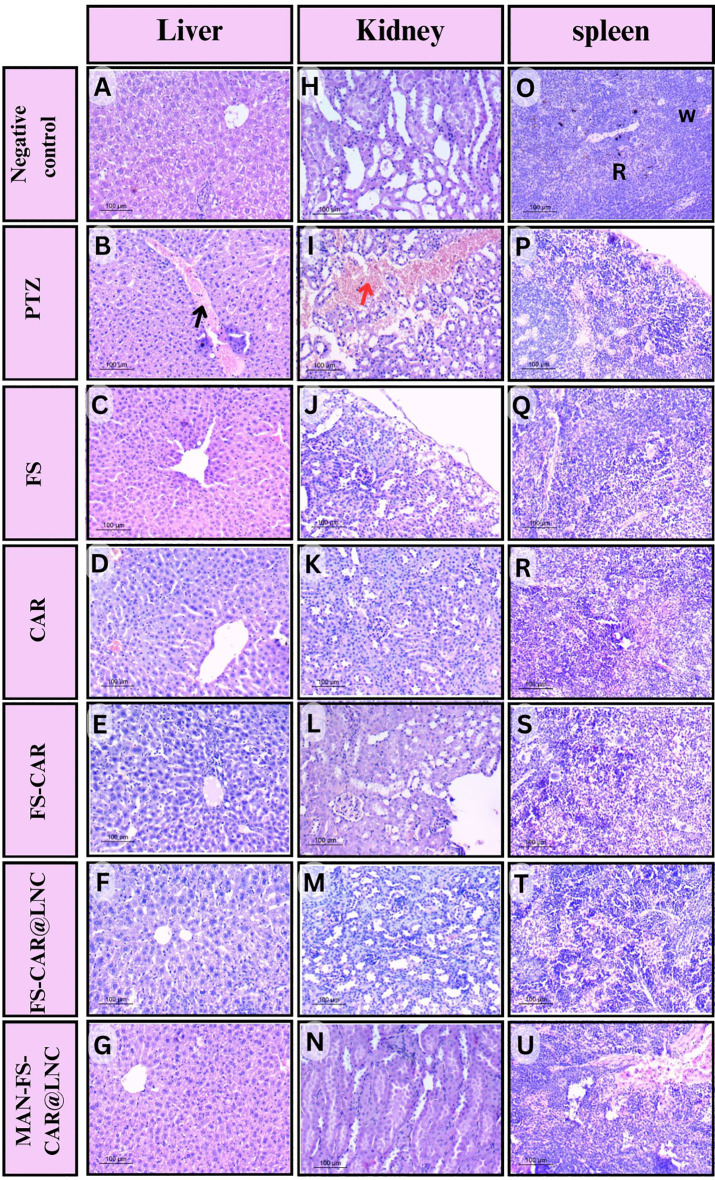
Representative H&E-stained organ samples of male rats, showing the liver (**A**- **G**); kidney (**H**- **N**) and spleen (**O**- **U**). (magnification X 200). Black arrow refers to congested dilated central vein, red arrow refers to hemorrhage, (R) refers to red pulp and (W) refers to white pulp

Potential toxicity that may arise from FS and CAR either free or loaded into LNC was evaluated by the histopathological inspectionof liver, kidney and spleen sections (Fig. 8) and biochemical analyses of liver and kidney functions (Table 4).

**Table 4:** Effect of FS, CAR, FS/CAR FS/CAR@LNC and MAN-FS/CAR@LNC on liver and kidney function tests

Group	ALT (U/mL)	AST (U/mL)	Urea (mg/dL)	Creatinine (mg/dL)
Healthy	54.67 ± 2.49	41 ± 5.35	41.67 ± 0.94	1 ± 0.14
PTZ group	51.86 ± 8.24	46.67 ± 6.24	42.6 ± 3.93	0.89 ± 0.18
FS	51 ± 2	36.75 ± 7.33	39.5 ± 3.64	0.9 ± 0.12
CAR	47.67 ± 8.24	37 ± 5.83	41.5 ± 1.5	0.86 ± 0.1
FS/CAR	52.4 ± 8.89	48.33 ± 4.78	40.8 ± 4.79	0.85 ± 0.18
FS/CAR@LNC	51.75 ± 3.49	49.5 ± 4.5	42.33 ± 3.09	0.9 ± 0.17
Man-FS/CAR@LNC	57 ± 4.97	40.4 ± 4.03	39.75 ± 3.27	0.93 ± 0.08

#### Histopathological study

Liver sections examined showed typical cytoarchitecture, with hepatocytes distributed in cords extending from the central vein. The hepatic sinusoids appeared as narrow gaps surrounded by flattened endothelial cells and scattered irregular kupffer cells with oval nuclei. In addition to the preceding findings, the PTZ group showed a dilated, congested central vein (Fig. 8B).

Renal architecture in all groups (Fig. 8) revealed typical Bowman’s capsules with subcapsular space. Many proximal and distal convoluted tubules were typically lined with epithelial tissue and contained by normal lamina. PTZ excretion in renal tissue caused structural damage, including numerous degenerative tubules (Fig. 8I). Structural damage disappeared in all treated groups, demonstrating the efficiency of FS [82] and CAR [83] against this toxicity condition.

Also, the splenic architecture was described as a well-defined white and red pulp with continuous trabecularity throughout the tissues (Fig. 8). Unlike the toxicity seen in liver and kidney in the PTZ group, spleens did not exhibit significant changes in structure (Fig. 8).

#### Serum biochemical analysis

All treated mice survived throughout the study. An insignificant change in body weight was observed (*p* > 0.05). Liver function was assessed by measuring AST and ALT while kidney function was monitored by investigating urea and creatinine serum levels(Table 4). An insignificant change in all groups comparable to the healthy group (*p* > 0.05) was recorded. Previous articles [82, 83] stated the safety and biocompatibility of LNC formulations [51].

## Conclusion

LNC co-loaded with the anti-inflammatory and antioxidant neuroprotective phytomedicines FS and CAR were prepared and evaluated in PTZ-induced chronic epilepsy model as a promising antiseizure treatment. LNC mannosylation (MAN-FS/CAR@LNC) for a further boost in brain bioavailability was firstly formulated and appraised in this study. Enhanced brain targetability was confirmed by both the IVIS of the fluorescent LNC (MAN-Cou-6@LNC) showing enhanced brain accumulation and the restoration of normal behavioral, biochemical and histopathological features of MAN-FS/CAR@LNC treated mice. All parameters assessed showed comparable observations to healthy mice. These findings present MAN-FS/CAR@/LNC as a promising nanoplatform with brain targeting potential circumventing chronic epilepsy and raising further research on other CNS disorders. In the current study MAN-FS/CAR@/LNC for brain targeting in kindling model were administered via IP route of administration as proof-of-concept. Testing brain targetability via oral route to confirm suitability of mannosylated LNC for human translation is highly encouraged in future research.

## Supplementary Information

Below is the link to the electronic supplementary material.

Supplementary Material 1

## Data Availability

The authors confirm that the data for this study findings are available within the article and the supplementary information file.
