# Supplementary material for: Mannosylated fisetin/carveol lipid nanocapsules: brain-targeted dual therapy for modulation of epileptogenesis and cognitive deficits
Source: Drug Deliv Transl Res. 2025 Aug 9;16(5):1349–71. doi: 10.1007/s13346-025-01937-2 (PMC13038668; doi:10.1007/s13346-025-01937-2)
Supplement: Supplementary file 1 — Supplementary Material 1 [file 13346_2025_1937_MOESM1_ESM.docx]

**Mannosylated Fisetin/Carveol Lipid Nanocapsules: Brain-Targeted Dual Therapy for Modulation of Epileptogenesis and Cognitive Deficits**

Julie R. Youssef ^1^, Nabila A. Boraie ^1^, Fatma A. Ismail^1^, Basant A. Bakr^2^, Eman A. Allam^3^, Mahmoud A. Agami ^4,5^ and Riham M. El-Moslemany^1^

^1^ Department of Pharmaceutics, Faculty of Pharmacy, Alexandria University, Alexandria 21521, Egypt

^2^ Department of Zoology, Faculty of Science, Alexandria University, Alexandria 21523, Egypt

^3^ Department of Medical Physiology, Faculty of Medicine, Alexandria University, Alexandria 21131, Egypt.

^4^ Faculty of pharmacy, New Valley University, New Valley Governorate, Egypt

^5^ Research and innovation hub, Alamein International University, New Alamein city, Egypt

Corresponding author: Riham M. El-Moslemany

Department of Pharmaceutics, Faculty of Pharmacy, Alexandria University.

1 Khartoum Square, Azarita, Messalla Post Office, P.O.Box 21521, Alexandria, Egypt.

Tel.: 002 01006020405

Email: [riham.elmoslemany@alexu.edu.eg](mailto:riham.elmoslemany@alexu.edu.eg)


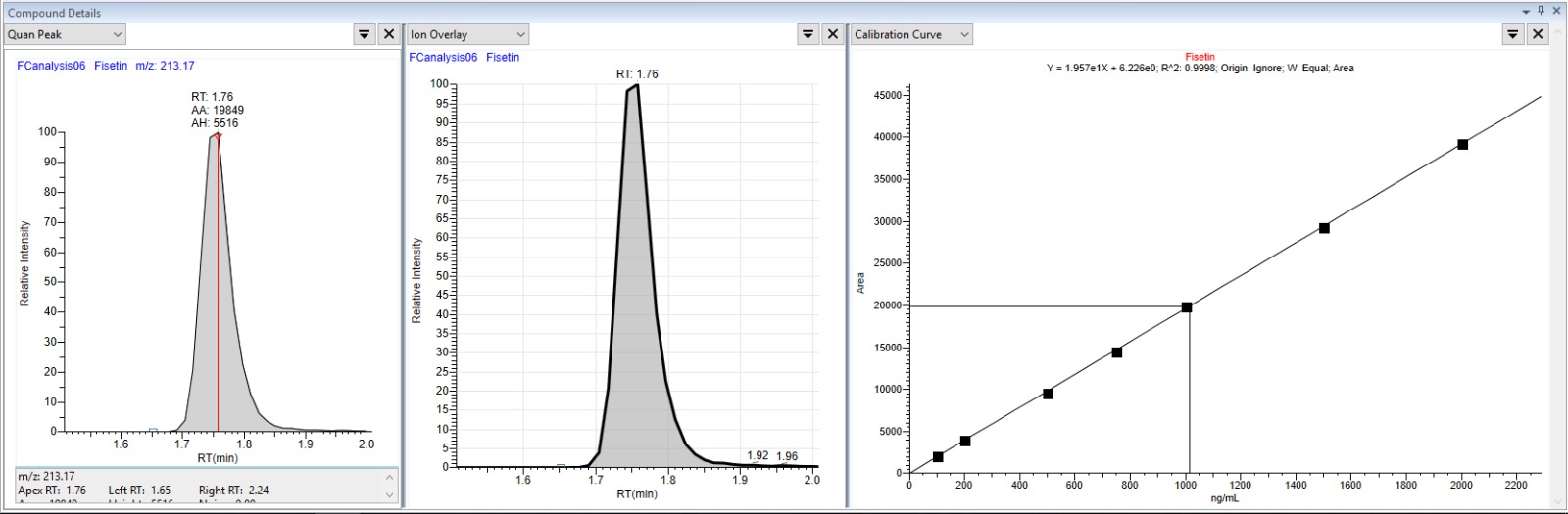


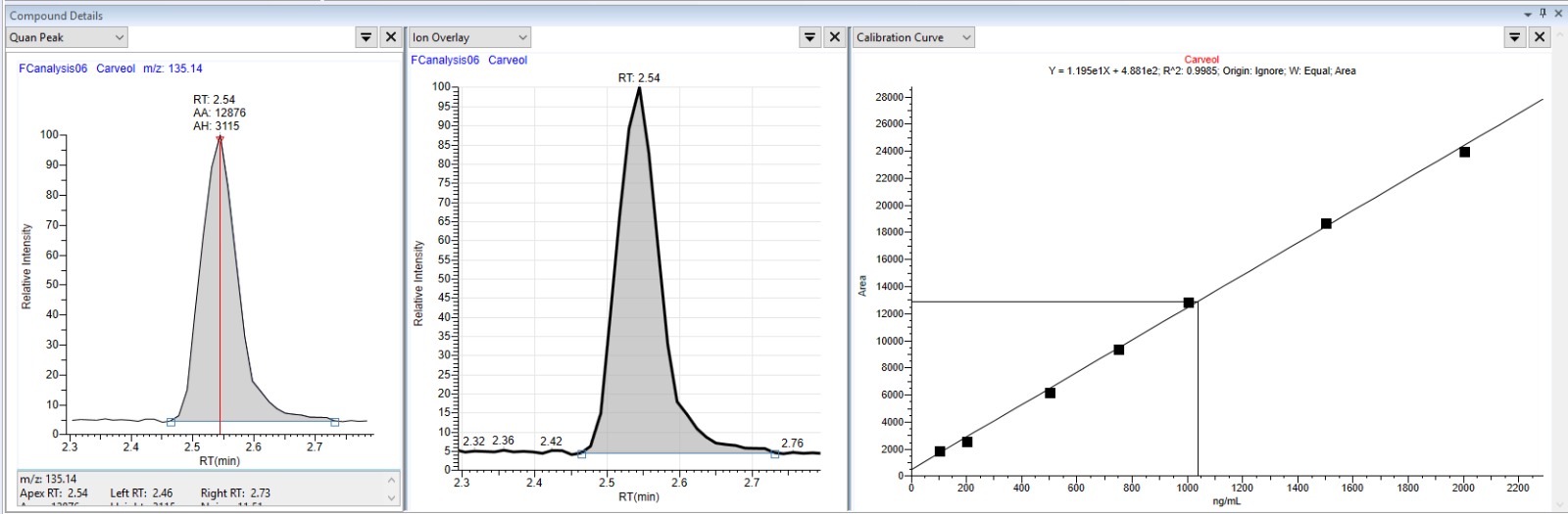


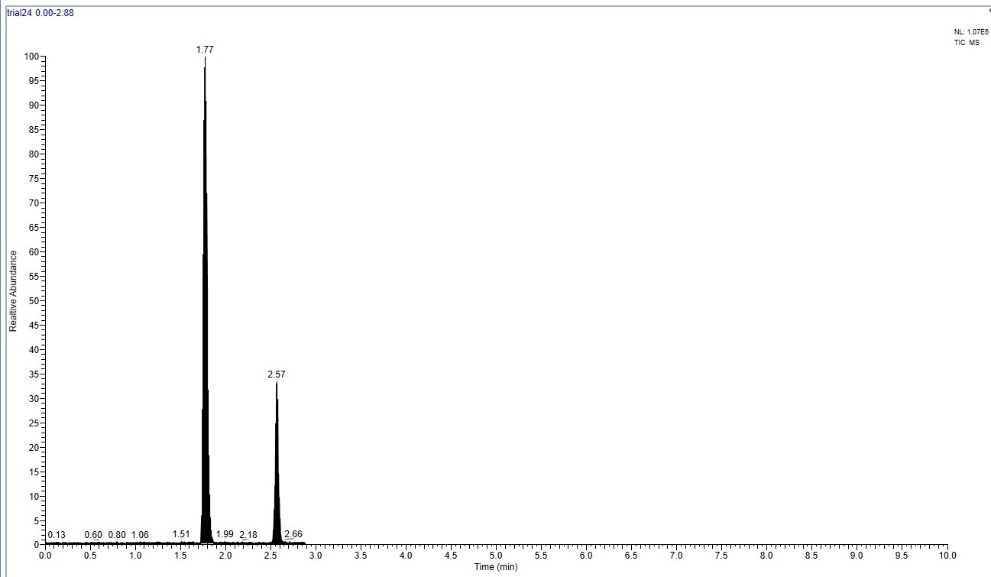


**Figure S1: Chromatograms and calibration curves for fisetin and carveol.**

**Table S1: Kinetic modeling of drug release from different LNC formulation**

|  | Fisetin | | | | | | Carveol | | | |
| --- | --- | --- | --- | --- | --- | --- | --- | --- | --- | --- |
| Formula | FS@LNC | | FS/CAR@LNC | | MAN-FS/CAR@LNC | | FS/CAR@LNC | | MAN-FS/CAR@LNC | |
|  | MSE | r | MSE | r | MSE | r | MSE | r | MSE | r |
| Zero-order | 172.48 | 0.691 | 138.42 | 0.692 | 77.787 | 0.69 | 440.3 | 0.699 | 184.1 | 0.748 |
| First-order | 158.76 | 0.71 | 128.85 | 0.707 | 73.599 | 0.708 | 366 | 0.747 | 161.9 | 0.776 |
| Higuchi | 63.78 | 0.839 | 50.935 | 0.839 | 27.456 | 0.848 | 154.67 | 0.85 | 56.1 | 0.88 |
| Hixon-crowell | 163.46 | 0.703 | 132.129 | 0.702 | 75.02 | 0.703 | 393.75 | 0.73 | 169.74 | 0.766 |
| Korsmeyer-peppas | 0.134 | 0.999 | 1.202 | 0.992 | 0.8773 | 0.9903 | 14.27 | 0.97 | 14.72 | 0.956 |
|  | n= 0.152 | | n= 0.158 | | n= 0.171 | | n= 0.199 | | n= 0.256 | |
